# Cost-effectiveness of short-protocol emergency brain MRI after negative non-contrast CT for minor stroke detection

**DOI:** 10.1007/s00330-021-08222-z

**Published:** 2021-08-28

**Authors:** Daniel Puhr-Westerheide, Matthias F Froelich, Olga Solyanik, Eva Gresser, Paul Reidler, Matthias P Fabritius, Matthias Klein, Konstantin Dimitriadis, Jens Ricke, Clemens C Cyran, Wolfgang G Kunz, Philipp M Kazmierczak

**Affiliations:** 1 Department of Radiology, University Hospital, LMU Munich, Marchioninistr. 15, 81377 Munich, Germany; 2 Department of Radiology and Nuclear Medicine, University Medical Centre Mannheim, Theodor-Kutzer-Ufer 1-3, 68167 Mannheim, Germany; 3 Department of Neurology, University Hospital, LMU Munich, Marchioninistr. 15, 81377 Munich, Germany; 4 Institute for Stroke and Dementia Research (ISD), University Hospital, LMU Munich, Munich, Germany

**Keywords:** Cost-effectiveness analysis, Ischemic stroke, Brain MRI, Secondary prevention, Quality-adjusted life years

## Abstract

**Objectives:**

To investigate the cost-effectiveness of supplemental short-protocol brain MRI after negative non-contrast CT for the detection of minor strokes in emergency patients with mild and unspecific neurological symptoms.

**Methods:**

The economic evaluation was centered around a prospective single-center diagnostic accuracy study validating the use of short-protocol brain MRI in the emergency setting. A decision-analytic Markov model distinguished the strategies “no additional imaging” and “additional short-protocol MRI” for evaluation. Minor stroke was assumed to be missed in the initial evaluation in 40% of patients without short-protocol MRI. Specialized post-stroke care with immediate secondary prophylaxis was assumed for patients with detected minor stroke. Utilities and quality-of-life measures were estimated as quality-adjusted life years (QALYs). Input parameters were obtained from the literature. The Markov model simulated a follow-up period of up to 30 years. Willingness to pay was set to $100,000 per QALY. Cost-effectiveness was calculated and deterministic and probabilistic sensitivity analysis was performed.

**Results:**

Additional short-protocol MRI was the dominant strategy with overall costs of $26,304 (CT only: $27,109). Cumulative calculated effectiveness in the CT-only group was 14.25 QALYs (short-protocol MRI group: 14.31 QALYs). In the deterministic sensitivity analysis, additional short-protocol MRI remained the dominant strategy in all investigated ranges. Probabilistic sensitivity analysis results from the base case analysis were confirmed, and additional short-protocol MRI resulted in lower costs and higher effectiveness.

**Conclusion:**

Additional short-protocol MRI in emergency patients with mild and unspecific neurological symptoms enables timely secondary prophylaxis through detection of minor strokes, resulting in lower costs and higher cumulative QALYs.

**Key Points:**

• *Short-protocol brain MRI after negative head CT in selected emergency patients with mild and unspecific neurological symptoms allows for timely detection of minor strokes.*

• *This strategy supports clinical decision-making with regard to immediate initiation of secondary prophylactic treatment, potentially preventing subsequent major strokes with associated high costs and reduced QALY.*

• *According to the Markov model, additional short-protocol MRI remained the dominant strategy over wide variations of input parameters, even when assuming disproportionally high costs of the supplemental MRI scan.*

## Introduction

Ischemic stroke is one of the leading causes of severe disability and a major cause for cardiovascular mortality worldwide [1–3]. The economic burden caused by ischemic strokes is very high, with about $65.5 billion spent on stroke in the USA in 2008 and an expected increase to about $184.1 billion in 2012 to 2030 [4]. Acute stroke care accounts for about one half of total direct medical costs within the first 12 months following ischemic stroke, and post-stroke care significantly contributes to the large expenses with rehabilitation services and facilities as key factors [4, 5]. In the acute setting, the length of stay in hospital is the most important cost driver [3]. Major strokes are preceded by minor strokes or transient ischemic attacks (TIA) in approximately 15–30%, with 40% of these precursor events occurring within 7 days and about 20% within 24 h before a major ischemic stroke [6, 7]. The definition of the term minor stroke is equivocal; however, it is commonly applied to ischemic strokes with a low National Institutes of Health Stroke Scale (NIHSS) score (≤ 1 or ≤ 3, respectively) [8]. Up to 65% of all acute ischemic events leading patients to seek medical attention are minor strokes or TIAs [9]. Urgent identification of these patients and immediate initiation of secondary prophylaxis lead to a risk reduction of 80 to 90% with regard to subsequent major strokes [9–11]. Therefore, timely diagnosis of minor strokes is of foremost importance for rapid initiation of secondary prophylaxis and prevention of major stroke.

In the majority of emergency departments, computed tomography (CT) of the head is the primary imaging modality in neurological patients with suspected intracranial pathology. In severely neurologically impaired patients with high pretest probability of ischemic stroke, non-contrast CT may be complemented by CT angiography of the extra- and intracranial vessels and whole-brain CT perfusion imaging [12, 13]. In small ischemic lesions, non-contrast CT may remain negative, especially in the acute phase [14–16]. However, the primary role of non-contrast CT in acute stroke is ruling out intracranial hemorrhage, as contraindication for intravenous thrombolytic therapy, not the detection of early ischemia signs. Depending on the overall clinical presentation, the likelihood of ischemic stroke, the absence of intracranial hemorrhage, and the time from symptom onset, the neurologist has three main options in these patients: (1) intravenous thrombolysis and admission to a stroke unit with further clinical workup (including electrocardiography and clinical risk assessment); (2) admission to a stroke unit, immediate initiation of secondary prophylaxis, and further clinical workup including MRI; (3) discharge from hospital after clinical risk assessment with or without secondary prophylaxis and referral to a neurological outpatient clinic for further clinical workup including MRI. However, time and monetary resources are known to limit the availability of MRI in the outpatient environment. In addition, a substantial percentage of neurological emergency patients present with mild and unspecific clinical symptoms and an overall low risk for ischemic stroke [17]. To detect small ischemic lesions and to increase diagnostic confidence in the emergency setting, it would be desirable to perform a short emergency MRI scan in this subset of selected patients. Vice versa, a negative MRI scan in the acute setting may support the diagnosis of stroke mimics and avoid unnecessary hospital admission and unnecessary secondary prophylaxis. In a previous study, short-protocol emergency MRI proved to be equivalent to standard-length protocol MRI with regard to image quality and lesion detection [18].

We hypothesized that short-protocol brain MRI is cost-effective for the detection of minor strokes in selected emergency patients with inconclusive neurological symptoms. The aim was to investigate the cost-effectiveness of supplemental short-protocol brain MRI after negative non-contrast CT in neurological emergency patients with mild and unspecific clinical symptoms.

## Materials and methods

**Fig. 1:**
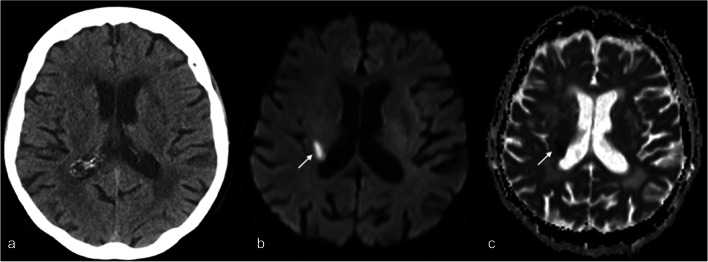
Representative CT-occult minor stroke in a patient presenting with transient left-sided mild hemiparesis. Non-contrast CT (**a**) did not show any signs of ischemic stroke. Diffusion-weighted imaging (**b**, b1000) and the corresponding apparent diffusion coefficient map (ADC map, **c**) from the short MRI protocol revealed an ischemic lesion in the posterior limb of the right internal capsule (arrow)

This study did not require institutional review board approval. The analysis was based on a previously published prospective single-center diagnostic accuracy study validating a vendor-specific brain MRI short-protocol (5 standard sequences: sagittal T1-weighted gradient echo, axial T2-weighted turbo spin echo, axial T2-weighted turbo spin echo fluid attenuated inversion recovery, axial-diffusion weighted single-shot echo-planar imaging, axial T2*-weighted echo-planar imaging gradient echo; total acquisition time including localizer 04:33 min) against a standard-length protocol (equivalent 5 standard sequences, total acquisition time including localizer 15:25 min) for use in the neurological emergency setting [18]. Four hundred forty-nine patients with acute non-traumatic neurological symptoms presenting to the emergency department of a large university center were screened for study inclusion. Of these, 60 patients with suspected intracranial pathology and negative non-contrast head CT were included and subsequently scanned with the short-protocol MRI and the standard-length protocol, which served as reference standard. The short-protocol MRI proved to be equivalent to the standard-length protocol with regard to image quality and the detection of intracranial pathologies (sensitivity 0.939 [0.881–0.972]; specificity 1.000 [0.895–1.000]). Compared to CT, 93 additional intracranial lesions were detected by short-protocol MRI, of which 21 were acute minor strokes, leading to a change in patient management (initiation of acetyl-salicylic acid as secondary prophylaxis, admission to dedicated stroke unit) in 10% (6/59). A representative patient with CT-occult minor stroke detected by short-protocol MRI is shown in Fig. 1.

### Model structure

#### Strategy modeling

**Fig. 2:**
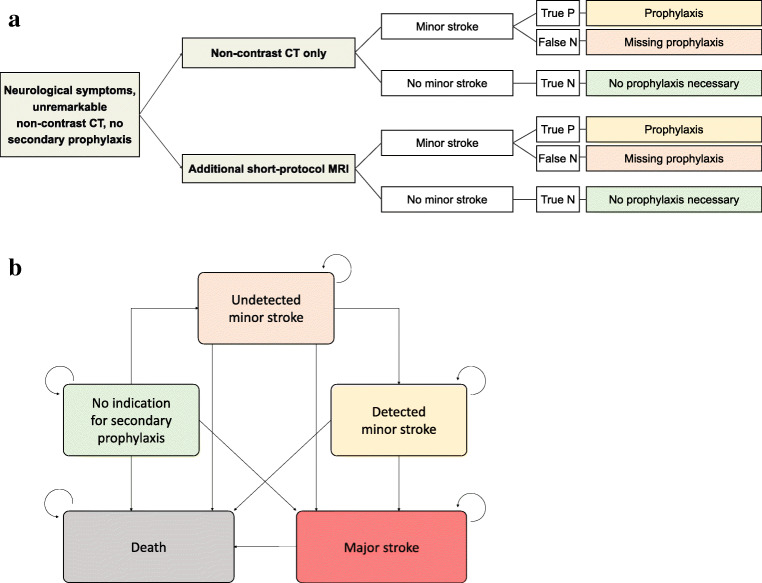
**a** Model overview. Comparison of standard strategy and employment of additional short-protocol MRI. True P, true positive; False N, false negative; True N, true negative. **b** Markov model. Long-term modeling of patient outcomes and long-term costs. Starting state defined by position in model

A decision model distinguishing the strategies “No additional imaging” and “Additional short-protocol MRI” was constructed (Fig. 2). In case of no additional imaging, a minor stroke was assumed to be missed in 40% of patients in the initial evaluation in the emergency situation. When applying “Additional short-protocol MRI,” based on the sensitivity, detection of a minor stroke was assumed in the majority of patients. In these cases, adequate post-stroke care with subsequent secondary stroke prophylaxis and regular office visits as well as additional long-term ECG monitoring was assumed including benefits in outcome due to appropriate medication and care [19].

#### Outcome modeling

For long-term evaluation, a Markov model was constructed (Fig. 2B). This model differentiated between patients without indication for secondary prophylaxis; patients with undetected minor stroke; patients with detected minor stroke, i.e. under adequate secondary prophylaxis; and those with major stroke. Mortality for every Markov state was included into the model. The model was run for 30 cycles, simulating a follow-up period of up to 30 years.

### Input parameters

All input parameters are summarized in Table 1.

**Table 1:** Input parameters

Input parameter	Estimate	Distribution	Source
Average age	61 years	–	18
Willingness to pay	$100,000	–	20–22
Discount rate	3.00%	–	23
Markov model time	Until death	–	-
Prevalence of minor stroke in collective	10.17%	–	18
Diagnostic performances			
No additional imaging sensitivity	60.00%	β	Assumption
No additional imaging specificity	100.00%	β	–
Additional ultrafast MRI sensitivity	94.00%	β	18
Additional ultrafast MRI specificity	100.00%	β	18
Acute diagnostic and treatment costs			
CT brain without contrast agent (Medicare code 76450)	$155	**γ**	24
Ultrafast MRI of brain (Medicare code 70551)	$309	**γ**	24
Acute care of minor stroke patient	$3000	**γ**	3,5
Acute care of major stroke patient	$6500	**γ**	3,5
Long-Term treatment costs			
Yearly costs of post-stroke care after mild stroke	$ 7023	**γ**	25,26
Yearly costs of post-stroke care after major stroke	$ 19,062	**γ**	25,26
Utilities			
QOL of patients without major stroke assuming mRS of 0	1.00	β	28
QOL of patients after major stroke assuming an average mRS of 4	0.33	β	28
Death	0.00	β	-
Transition probabilities: risk of death			
Risk of death without indication for secondary prophylaxis	US life tables	β	29
Additional risk of death with undetected minor stroke	0.075	β	11,30
Additional risk of death with detected minor stroke under secondary prophylaxis	0.06	β	30
Relative risk of death after major stroke assuming an average mRS of 4	1.71	β	29,31
Transition probabilities: risk of stroke			
Risk of new minor stroke	0.003	β	9,32,33
Yearly detection rate of minor stroke	0.1	β	Assumption
Risk of major stroke in patients without minor stroke	0.006	β	32,33
Risk of major stroke with undetected minor stroke (first year) assuming 80% risk reduction with secondary preventive treatment	0.255	β	11,30,34
Risk of major stroke with undetected minor stroke (after first year) assuming 80% risk reduction with secondary preventive treatment	0.055	β	11,30,34
Risk of major stroke with detected minor stroke (first year)	0.051	β	30
Risk of major stroke with detected minor stroke (after first year)	0.011	β	34

#### Modeling parameters

Willingness to pay was set to $100,000 per QALY according to international recommendations [20–22]. In addition, a discount rate of 3% was applied for both costs and quality of life [23].

#### Diagnostic performance parameters and pretest probability

Based on the above-referenced diagnostic accuracy study, diagnostic sensitivity was set to 94% and specificity to 100% [18]. Prevalence of minor stroke was assumed in 10.17% of cases in the collective with an average age of 61 years [18]. As only patients without CT signs of stroke were included, the sensitivity of the strategy without short-protocol MRI was set to 60% based on clinical assessment leading to minor stroke diagnosis. As no false positives could occur in this setting, the specificity was set to 100%.

#### Short-term diagnostic costs

Costs of non-contrast CT were set to $155 based on Medicare Procedure Code 70450 [24]. Additional costs of non-contrast MRI were assumed as $309 according to Medicare Procedure Code 70551 [24]. Since a price reduction of short-protocol MRI is not established yet, our model was calculated based on the full price of a normal-length protocol as described above.

#### Short-term treatment costs

Expected average acute treatment costs for minor and major stroke were obtained from the literature and set to $3000 and $6500, respectively [3, 5].

#### Long-term costs

No additional costs for patients with undetected minor stroke were assumed. Treatment costs for secondary prophylaxis including doctor visits were estimated as $7023 per year based on published literature [25, 26]. Yearly costs after major stroke were set to $19,062 per year based on previously published literature on economic implications of ischemic stroke [26, 27].

#### Utilities

Utilities and quality-of-life measures were estimated as quality-adjusted life years (QALYs). Utilities of patients after a minor, undetected stroke were assumed to be not reduced. The quality of life of patients after a major stroke was set to 0.33 assuming an average disability equal to a modified Rankin Scale (mRS) of 4 [28].

#### Transition probabilities for risk of death

Average, age-specific risk of death was extracted from US life tables and assumed for patients without indication for secondary prophylaxis and without history of stroke [29]. An increased risk of death for patients with undetected minor stroke, with detected minor stroke under secondary prophylaxis and relative risk of death after major stroke were extracted from literature [11, 29–31].

#### Transition probabilities for risk of stroke

The yearly risk of a newly occurring minor stroke without the presence of risk factors was set to 0.3% [9, 32, 33]. Given that studies on yearly detection rates of undetected minor strokes are not present, the yearly detection rate of undetected strokes was set to 10% based on assumptions. However, wide variations of this input parameter were analyzed in the sensitivity analysis. The risk of a major stroke in the general population has been reported as about 0.6% per year [32, 33], whereas this value is increased significantly after a minor stroke under secondary preventive treatment at about 5.1% in the first year [30] and 1.1% in the following years [34]. Based on this information, the risk of a major stroke in patients without prophylactic treatment was calculated based on a risk reduction of 80% due to prophylactic treatment [11, 30, 34].

### Cost-effectiveness analysis

The analysis was performed using dedicated decision-modeling software (TreeAge Pro Healthcare 2020).

### Sensitivity analysis

To investigate the model stability with respect to its input parameters, two sensitivity analyses were carried out: First, a deterministic sensitivity analysis varying individual input parameters (costs, utilities, diagnostic accuracies, etc.) was carried out in order to compare the univariable impact of the parameters on the model outputs. Second, a probabilistic sensitivity analysis including 30,000 Monte Carlo iterations was carried out to investigate the overall model stability and variation of outputs.

## Results

### Base case analysis

In the base case analysis including all input parameters from Table 1 and a modeling time of 30 years, the CT-only strategy resulted in overall costs of $27,109 compared to $26,304 in the additional short-protocol MRI group. The cumulative calculated effectiveness in the CT-only group was approximately 14.25 QALYs compared to 14.31 QALYs in the additional short-protocol MRI group. As a result, additional abbreviated MRI was the dominant strategy in this patient collective. These results are shown in Table 2.

**Table 2:** Base case analysis results

Strategy	Cumulative costs	Cumulative effectiveness	Interpretation
CT only	$27,109	14.25 QALYs	Dominated strategy
Additional short-protocol MRI	$26,304	14.31 QALYs	Dominant strategy
Delta	− $805	0.06 QALYs	

### Deterministic sensitivity analysis

In the deterministic sensitivity analysis, additional short-protocol MRI remained the dominant strategy in all ranges investigated.

In detail, the additional risk of major stroke after the first year following detection of minor stroke had the most significant impact on the cost-effectiveness of additional short-protocol MRI. Although higher age reduced the relative cost-effectiveness of additional short-protocol MRI, it remained the dominant strategy in the range investigated, i.e., 50 to 70 years. In comparison, the quality of life after major stroke had a lower impact on cost-effectiveness than risk measures for major stroke before and after minor stroke. Also, additional short-protocol MRI remained the dominant strategy in the ranges investigated. Even when assuming a relatively high cost of abbreviated MRI of $500, it remained the cost-effective strategy.

**Fig. 3:**
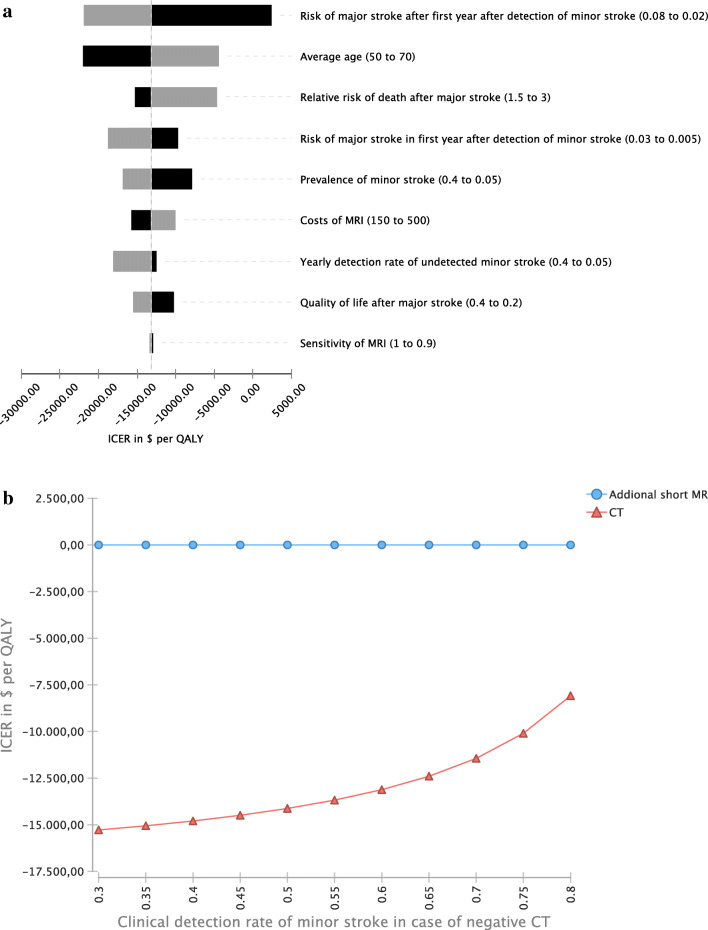
**a** Deterministic sensitivity analysis. In all ranges investigated, additional short-protocol MRI remained the dominant strategy. **b** Incremental cost-effectiveness ratio (ICER). ICER are presented depending on clinical detection rates of minor strokes in patients not undergoing short-protocol MRI. MRI remains the cost-effective strategy in all investigated ranges

In summary, transition probabilities, detection rates, and quality of life after a major stroke had a higher influence on the cost-effectiveness of additional short-protocol MRI than costs and sensitivity of MRI. The results are shown as a tornado plot in Fig. 3A. Further, additional short-protocol MRI remained the dominant strategy even when assuming a rate of missed minor strokes of as low as 20% in patients not undergoing short-protocol MRI (Fig. 3B).

### Probabilistic sensitivity analysis

**Fig. 4:**
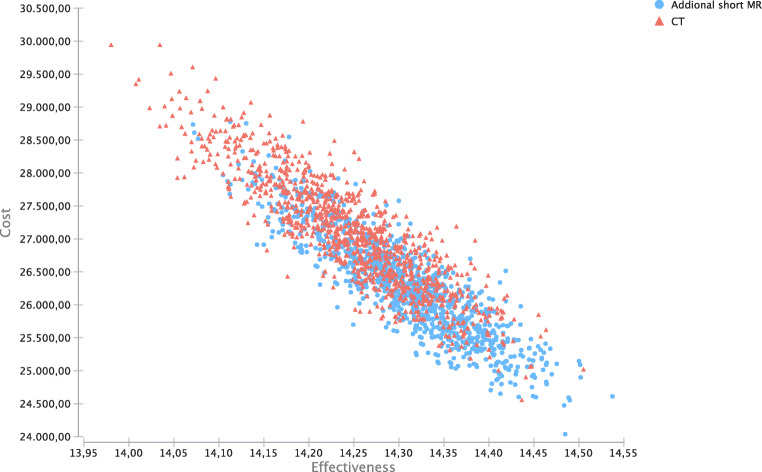
Probabilistic sensitivity analysis for both strategies. Additional short-protocol MRI in blue, no additional imaging in red

Results from the base case analysis were confirmed in the probabilistic sensitivity analysis, with “Additional short-protocol MRI” resulting in on average lower costs and higher effectiveness (Fig. 4).

## Discussion

The present analysis reveals short-protocol MRI as a cost-effective strategy in emergency patients with suspected intracranial pathology and negative non-contrast head CT presenting with inconclusive neurological symptoms, being dominant even from an economic perspective. In this setting, the cost-savings due to a lower rate of major strokes and gains in effectiveness by application of preventive treatment may by far outweigh the additional costs of supplemental short-protocol MRI subsequent to negative head CT in the emergency situation.

Major strokes are preceded by minor strokes or transient ischemic attacks (TIA) in 15–30%, and about 40% of recurrent major strokes occur within 7 days and about 20% within 24 h after the initial minor stroke or TIA [6, 7, 30]. Urgent initiation of secondary prophylactic treatment after a minor stroke or TIA was demonstrated to prevent 80 to 90% of recurrent major strokes [9–11]. The importance of a rapid treatment start has been demonstrated in detail in the EXPRESS study with a rate of recurrent stroke of 2.1% in patients receiving treatment within 1 day of the index event compared to a rate 10.3% in patients receiving treatment within 3 days of the index event [11]. These data clearly point to the pivotal role of an early detection of minor strokes, a current diagnostic gap that may be closed with MRI in the acute setting [14–16].

Major stroke is of utmost clinical and economical importance, as it is associated with a very high economic burden for healthcare systems. About 40% of all stroke patients suffer moderate to severe disabilities and need special care; about 10% depend on long-term care facilities [4]. Acute stroke care accounts for about one half of total direct medical costs within the first 12 months following ischemic stroke, and post-stroke care significantly contributes to the large expenses. Rehabilitation services and facilities represent the most expensive factors [4, 5]. In the acute setting, the length of stay in hospital is the most important cost driver [35]. Minor stroke or TIA patients have a significantly shorter length of stay in hospital, less patients have to be admitted to the intensive care unit, and less patients are discharged to skilled nursing facilities with consecutive high expenses [3, 5].

In this context, our results indicate that additional investment in short-protocol MRI examinations in the emergency setting does not only increase the detection rate of minor strokes, but can be regarded as a highly cost-effective, dominant strategy by preventing major strokes with consecutively high costs. In our analysis, we found overall higher costs for patients without additional short-protocol MRI compared to patients undergoing additional MRI. Further, the cumulative calculated effectiveness in the CT-only group was lower compared to the additional short-protocol MRI group. In our deterministic sensitivity analysis, additional short-protocol MRI remained the dominant strategy in the ranges investigated, and even when assuming a relatively high cost of the ultrafast MRI of $500, it remained the cost-effective strategy. Transition probabilities, detection rates, and quality of life after a major stroke had a higher influence on the cost-effectiveness of additional short-protocol MRI than costs and sensitivity of MRI itself.

Our study adds to the field as it demonstrated the cost-effectiveness of supplemental short-protocol MRI subsequent to negative head CT in neurological emergency patients. In many related fields of research, the additional utilization of procedures and diagnostic tests has been shown to be cost-effective: Despite high costs in the acute setting, interventional treatment has been shown to be cost-effective in major stroke patients, even in patients with a limited overall life expectancy [26, 36]. Furthermore, a transition from a time window to a tissue window for stroke patients presenting with unknown onset time for endovascular treatment and intravenous thrombolysis has taken place [37–39]. In this context, higher costs of MRI have been found to be cost-effective for major stroke patients with unknown onset time in order to allow treatment beyond traditional time windows in selected patients with salvageable brain tissue, thereby improving functional outcome after major stroke [40]. The cost-effectiveness of secondary prophylaxis, e.g., with antiplatelet therapy, has also been demonstrated in previous studies [41–43].

In future studies and cost-effectiveness analyses, initial imaging with short-protocol or standard-length MRI instead of CT for selected patients should be investigated, as this may lead to an optimized diagnosis and treatment planning in patients with minor stroke symptoms.

### Limitations

Yet, results of this cost-effectiveness evaluation have to be interpreted in the context of certain limitations:

First, input parameters are key factors for the modeling of cost-effectiveness evaluations. Still, these input parameters are derived from published literature and their data basis can vary between input variables. Further, the yearly detection rate of minor strokes or TIA had to be assumed since data on this parameter were not available and the number of patients with mild and unspecific symptoms and missed minor strokes discharged from the emergency department without secondary prophylaxis is not known and therefore had to be estimated. On the other hand, deterministic sensitivity analysis with wide ranges for input parameters allowed to model a variety of scenarios with all of them remaining cost-effective for additional short-protocol MRI in patients with neurological symptoms and inconclusive non-contrast head CT.

Second, every cost-effectiveness modeling has limitations due to the model structure. Although the Markov model applied takes into account several clinical situations, there may be special cases not fully reflected in the model. However, due to a tradeoff between model complexity and availability of (published) data, the authors believe that the current modeling approach may represent an acceptable middle ground in this respect.

Third, post-stroke care costs used by Earnshaw et al and Kunz et al are based on the stroke treatment economic model from 1996 [25, 26]. However, detailed and more recent data on post-stroke care costs from the USA for this particular case were not available. Acute stroke care costs increased disproportionately due to novel medical and mechanical treatment options. For these costs, more recent data were available.

Fourth, the rate of minor strokes in our cohort in selected emergency patients presenting with inconclusive neurological symptoms was based on study results from a prospective single-center diagnostic accuracy study. Minor stroke rates might differ slightly in large-scale multi-centric studies in this patient subpopulation. However, short-protocol MRI remained the dominant strategy in wide ranges for this input parameter assessed with our deterministic sensitivity analysis.

Fifth, our analysis was based on the US healthcare system. Cost-effectiveness might differ substantially between countries, and our results may not be transposed to other health care systems without adjustments and modifications.

## Conclusions

Additional short-protocol emergency brain MRI after negative non-contrast head CT is a cost-effective strategy in selected neurological patients with mild and unspecific symptoms, resulting in lower costs and higher QUALYs.

## Abbreviations

CT: Computed tomography
MRI: Magnetic resonance imaging
mRS: Modified Rankin Scale
NIHSS: National Institutes of Health Stroke Scale
QALY: Quality-adjusted life years
TIA: Transient ischemic attacks

## Declarations

### Guarantor

The scientific guarantor of this publication is Daniel Puhr-Westerheide.

### Statistics and biometry

One of the authors has significant statistical expertise.

### Methodology

• Performed at one institution

## References

[1] Thrift AG, Cadilhac DA, Thayabaranathan T (2014). Global stroke statistics. Int J Stroke.

[2] Wang H, Naghavi M, Allen C (2016). Global, regional, and national life expectancy, all-cause mortality, and cause-specific mortality for 249 causes of death, 1980–2015: a systematic analysis for the Global Burden of Disease Study 2015. Lancet.

[3] Buisman LR, Tan SS, Nederkoorn PJ (2015). Hospital costs of ischemic stroke and TIA in the Netherlands. Neurology.

[4] Rajsic S, Gothe H, Borba HH (2019). Economic burden of stroke: a systematic review on post-stroke care. Eur J Health Econ.

[5] Reed SD, Blough DK, Meyer K, Jarvik JG (2001). Inpatient costs, length of stay, and mortality for cerebrovascular events in community hospitals. Neurology.

[6] Rothwell PM, Warlow CP (2005). Timing of TIAs preceding stroke: time window for prevention is very short. Neurology.

[7] Rothwell PM, Giles MF et al (2005) A simple score (ABCD) to identify individuals at high early risk of stroke after transient ischaemic attack. Lancet. 8;366(9479):29–36. 10.1016/S0140-6736(05)66702-5 10.1016/S0140-6736(05)66702-5 15993230

[8] Fischer U, Baumgartner A, Arnold M (2010). What is a minor stroke?. Stroke.

[9] von Weitzel-Mudersbach P, Andersen G, Hundborg HH, Johnsen SP (2013). Transient ischemic attack and minor stroke are the most common manifestations of acute cerebrovascular disease: a prospective, population-based study--the Aarhus TIA study. Neuroepidemiology.

[10] Rothwell PM, Algra A, Chen Z (2016). Effects of aspirin on risk and severity of early recurrent stroke after transient ischaemic attack and ischaemic stroke: time-course analysis of randomised trials. Lancet.

[11] Rothwell PM, Giles MF, Chandratheva A (2007). Effect of urgent treatment of transient ischaemic attack and minor stroke on early recurrent stroke (EXPRESS study): a prospective population-based sequential comparison. Lancet.

[12] Thierfelder KM, Sommer WH, Baumann AB (2013). Whole-brain CT perfusion: reliability and reproducibility of volumetric perfusion deficit assessment in patients with acute ischemic stroke. Neuroradiology.

[13] Thierfelder KM, von Baumgarten L, Baumann AB (2014). Penumbra pattern assessment in acute stroke patients: comparison of quantitative and non-quantitative methods in whole brain CT perfusion. PLoS One.

[14] Moreau F, Asdaghi N, Modi J (2013). Magnetic resonance imaging versus computed tomography in transient ischemic attack and minor stroke: the more υou see the more you know. Cerebrovasc Dis Extra.

[15] Coutts SB, Simon JE, Eliasziw M (2005). Triaging transient ischemic attack and minor stroke patients using acute magnetic resonance imaging. Ann Neurol.

[16] Chaturvedi S, Ofner S, Baye F (2017). Have clinicians adopted the use of brain MRI for patients with TIA and minor stroke?. Neurology.

[17] Rizos T, Jüttler E, Sykora M (2011). Common disorders in the neurological emergency room - experience at a tertiary care hospital: common disorders in a specialized neurological ER. Eur J Neurol.

[18] Kazmierczak PM, Dührsen M, Forbrig R (2020). Ultrafast brain magnetic resonance imaging in acute neurological emergencies: diagnostic accuracy and impact on patient management. Invest Radiol.

[19] Kernan WN, Ovbiagele B, Black HR (2014). Guidelines for the prevention of stroke in patients with stroke and transient ischemic attack: a guideline for healthcare professionals from the American Heart Association/American Stroke Association. Stroke.

[20] Cameron D, Ubels J, Norström F (2018) On what basis are medical cost-effectiveness thresholds set?. Clashing opinions and an absence of data: a systematic review. Glob Health Action 11. 10.1080/16549716.2018.1447828 10.1080/16549716.2018.1447828 PMC5930346 29564962

[21] Neumann PJ, Cohen JT, Weinstein MC (2014). Updating cost-effectiveness — the curious resilience of the $50,000-per-QALY threshold. N Engl J Med.

[22] Woods B, Revill P, Sculpher M, Claxton K (2016). Country-level cost-effectiveness thresholds: initial estimates and the need for further research. Value Health.

[23] Sanders GD, Neumann PJ, Basu A (2016). Recommendations for conduct, methodological practices, and reporting of cost-effectiveness analyses. JAMA.

[24] Procedure price lookup for outpatient services | Medicare.gov. https://www.medicare.gov/procedure-price-lookup/. Accessed 25 Oct 2020

[25] Earnshaw SR, Dan J, Ray F, Lee S (2009). Cost-effectiveness of patient selection using penumbral-based MRI for intravenous thrombolysis. Stroke.

[26] Kunz WG, Hunink MGM, Sommer WH (2016). Cost-effectiveness of endovascular stroke therapy: a patient subgroup analysis from a US healthcare perspective. Stroke.

[27] Earnshaw SR, Jackson D, Farkouh R, Schwamm L (2009). Cost-effectiveness of patient selection using penumbral-based MRI for intravenous thrombolysis. Stroke.

[28] Napasri C, Opeolu A, Lewis RJ (2015). Adopting a patient-centered approach to primary outcome analysis of acute stroke trials using a utility-weighted modified Rankin scale. Stroke.

[29] Arias E (2018) Xu J. United States Life Tables, 2015. Natl Vital Stat Rep 67(7):1–64. PMID: 30707669 30707669

[30] Amarenco P, Lavallée PC, Labreuche J (2016). One-year risk of stroke after transient ischemic attack or minor stroke. N Engl J Med.

[31] Samsa GP, Reutter RA, Parmigiani G (1999). Performing cost-effectiveness analysis by integrating randomized trial data with a comprehensive decision model: application to treatment of acute ischemic stroke. J Clin Epidemiol.

[32] D’Agostino RB, Wolf PA, Belanger AJ, Kannel WB (1994). Stroke risk profile: adjustment for antihypertensive medication. The Framingham Study. Stroke.

[33] Dufouil C, Beiser A, McLure LA (2017). Revised Framingham stroke risk profile to reflect temporal trends. Circulation.

[34] Amarenco P, Lavallée PC, Monteiro Tavares L (2018). Five-year risk of stroke after TIA or minor ischemic stroke. N Engl J Med.

[35] Buisman LR, Tan SS, Nederkoorn PJ (2015). Hospital costs of ischemic stroke and TIA in the Netherlands. Neurology.

[36] Kunz WG, Hunink MG, Dimitriadis K (2018). Cost-effectiveness of endovascular therapy for acute ischemic stroke: a systematic review of the impact of patient age. Radiology.

[37] Nogueira RG, Jadhav AP, Haussen DC (2018). Thrombectomy 6 to 24 hours after stroke with a mismatch between deficit and infarct. N Engl J Med.

[38] Thomalla G, Simonsen CZ, Boutitie F (2018). MRI-guided thrombolysis for stroke with unknown time of onset. N Engl J Med.

[39] Albers GW, Marks MP, Kemp S (2018). Thrombectomy for stroke at 6 to 16 hours with selection by perfusion imaging. N Engl J Med.

[40] Pandya A, Eggman AA, Kamel H (2016). Modeling the cost effectiveness of neuroimaging-based treatment of acute wake-up stroke. PLoS One.

[41] Matchar DB, Samsa GP, Liu S (2005). Cost-Effectiveness of antiplatelet agents in secondary stroke prevention: the limits of certainty. Value Health.

[42] Malinina D, Zema C, Sander S, Serebruany V (2007). Cost–effectiveness of antiplatelet therapy for secondary stroke prevention. Expert Rev Pharmacoecon Outcomes Res.

[43] Rothlisberger JM, Ovbiagele B (2015). Antiplatelet therapies for secondary stroke prevention: an update on clinical and cost–effectiveness. J Comp Eff Res.

